# The ins and outs of liver fat metabolism: The effect of phenotype and diet on risk of intrahepatic triglyceride accumulation

**DOI:** 10.1113/EP092001

**Published:** 2025-01-24

**Authors:** Kieran Smith, Kaitlyn M. J. H. Dennis, Leanne Hodson

**Affiliations:** ^1^ Oxford Centre for Diabetes, Endocrinology and Metabolism University of Oxford Oxford UK; ^2^ Oxford NIHR Biomedical Research Centre Churchill Hospital Oxford UK

**Keywords:** dietary macronutrients, fatty acids, hepatic, human physiology, liver fat, phenotype

## Abstract

In health, the liver is a metabolically flexible organ that plays a key role in regulating systemic lipid and glucose concentrations. There is a constant flux of fatty acids (FAs) to the liver from multiple sources, including adipose tissue, dietary, endogenously synthesized from non‐lipid precursors, intrahepatic lipid droplets and recycling of triglyceride‐rich remnants. Within the liver, FAs are used for triglyceride synthesis, which can be oxidized, stored or secreted in very low‐density lipoproteins into the systemic circulation. The processes of FA uptake, FA synthesis and the intracellular partitioning of FAs into storage, oxidation or secretory pathways are tightly regulated. An imbalance in these processes causes intrahepatic triglyceride to accumulate and is associated with the development of metabolic dysfunction‐associated steatotic liver disease. It is well appreciated that many factors can influence intrahepatic FA partitioning, and although there is good evidence that both phenotype (e.g., sex, ethnicity and adiposity) and dietary macronutrient composition can play a role in intrahepatic triglyceride accumulation, their interaction remains poorly understood. The aim of this review is to explore how the respective pathways of FA delivery, synthesis and disposal are altered by phenotype and understand how dietary macronutrient composition might influence the partitioning of FAs in the liver in vivo, in humans.

## Abstract (highlights)

- **What is the topic of the review?**
  This review explores how fatty acid delivery, synthesis and disposal are altered by the phenotype of an individual and how dietary macronutrient composition can influence the hepatic partitioning of fatty acids in vivo in humans.
- **What advances does it highlight?**
  Studies using stable‐isotope tracers have delineated some of the complexity of how the phenotype of an individual and dietary composition can alter fatty acid delivery to, synthesis within and removal of fat from the liver. However, the interaction between diet and individual phenotype and the effect on intrahepatic fatty acid partitioning remains an area where work is sparse.

## 1 INTRODUCTION

The liver is the largest and one of the major metabolic internal organs in the human body. It is a homeostatic regulator of systemic lipid and glucose metabolism and demonstrates a high degree of metabolic flexibility, which allows it to transition rapidly between the metabolic tasks of energy storage and supply in response to fluctuations in energy demands (Hodson & Gunn, 2019).

### 1.1 Anatomical overview of the human liver

By sitting at the metabolic crossroads, the liver serves as an intermediary between exogenous and endogenous sources of energy and the extrahepatic organs that require energy. The liver is composed primarily of two lobes and is connected to the gut via the portal vein, which provides ∼75% of the total blood supply, with the remainder derived from the hepatic artery, which delivers oxygenated blood from the systemic circulation (Trefts et al., 2017). The portal vein, the hepatic artery and the bile duct (known as the portal triad) play a key role in hepatic substrate delivery. For example, medium chain fatty acids (FAs), amino acids (AAs) and glucose enter the liver via the portal vein, and chylomicron remnants, long‐chain FAs, lactate, pyruvate and AAs enter via the hepatic artery (Trefts et al., 2017). After substrates have undergone metabolic conversions, the respective products [e.g., CO_2_, very‐low density lipoprotein (VLDL), ketones and urea] are secreted into the hepatic venous drainage (from the left, right and middle hepatic veins) and into systemic circulation (Trefts et al., 2017).

Within the liver, hepatocytes are the main parenchyma of the liver, constituting ∼80% of tissue, with Kupffer cells, stellate cells, sinusoidal endothelial cells and cholangiocytes making smaller contributions (Ben‐Moshe & Itzkovitz, 2019). Each lobe is made up of lobules that consist of plates of hepatocytes and sinusoids distributed along the periportal–central axis (Porat‐Shliom, 2024); depending on their proximity to the portal vein or the hepatic artery, they are defined as periportal or pericentral, respectively. Hepatocytes in the periportal zone are exposed to the highest supply of nutrients, with nutrient concentrations declining progressively towards the pericentral zone (Porat‐Shliom, 2024). Given that hepatocytes are the main drivers of hepatic metabolic activity (Green et al., 2015), hepatocyte cellular integrity and function are important for the maintenance of metabolic health (Hodson & Gunn, 2019).

### 1.2 Overview of human liver FA metabolism

In health, the liver demonstrates metabolic plasticity and is able to shift rapidly between anabolic and catabolic processes in response to altered nutrient flux (e.g., during the transition from the fasted to fed state) and in response to nutritional and hormonal needs (Samuel & Shulman, 2016). Insulin plays a fundamental role in this regard by altering the delivery of substrates (e.g., FAs, glycerol, glucose and lactate) to the liver and by shifting hepatocellular metabolism away from energy utilization and towards energy storage [i.e., the conversion of glucose to glycogen and FAs to triglyceride (TG), and the attenuation of hepatic glucose production] by affecting post‐translational events (e.g., phosphorylation and dephosphorylation) (Hodson & Gunn, 2019; Samuel & Shulman, 2016).

Each day, the liver processes large quantities of FA (∼100 g/day; Frayn et al., 2006) from multiple sources, including non‐esterified fatty acids (NEFAs) that are liberated from adipose TG lipolysis and TG‐rich remnants [i.e., chylomicron remnants (in the fed state) and the continual recycling of VLDL‐TG]. Within the hepatocyte, FAs can also be synthesized from non‐lipid precursors, including monosaccharides (glucose and fructose) or AAs, via *de novo* lipogenesis (DNL). The majority of FAs within the liver are broadly partitioned between two pathways: esterification to form predominantly glycerolipids (TG and phospholipids) or mitochondrial oxidation, with the channelling into these respective pathways being dependent on both nutritional state and metabolic health (Hodson & Frayn, 2011).

It is suggested that an imbalance between the delivery of FAs to the liver, synthesis of FAs within the liver and their disposal from the liver causes intrahepatic TG (IHTG) to accumulate (Diraison & Beylot, 1998; Sidossis et al., 1998). The net retention of IHTG has pathological consequences and is closely associated with the metabolic syndrome, cardiovascular disease and type 2 diabetes (T2D) (Targher et al., 2024). Although there is good evidence demonstrating that the phenotype (age, ethnicity, adiposity and sex) and genetics of an individual, along with dietary macronutrient composition, might play a role in IHTG accumulation (Marjot et al., 2020), very few reports have investigated the underlying mechanisms driving alterations in IHTG content. The sparsity in the literature is attributable, in part, to the challenging nature of investigating hepatic metabolism in vivo in humans.

Therefore, the aim of this review is to explore how the respective pathways of FA delivery, synthesis and disposal are altered by phenotype and understand how dietary macronutrient composition can influence the partitioning of FAs in the liver in vivo, in humans. Although this review focuses on findings in humans, preclinical murine models and in vitro cell‐line models have been shown to recapitulate some aspects of human hepatic metabolism that are not possible to do in vivo in humans (Gunes & Estall, 2024). Thus, to provide mechanistic evidence to support the findings reported in humans, we also provide, when available, evidence from preclinical murine models.

## 2 INTRAHEPATIC TRIGLYCERIDE ACCUMULATION

Alterations in hepatic FA partitioning is a hallmark of IHTG accumulation (Fabbrini et al., 2010) and the pathological accumulation of IHTG, when unrelated to excessive alcohol consumption, is the initial step in metabolic dysfunction‐associated steatotic liver disease [(MASLD); previously known as non‐alcoholic fatty liver disease (NAFLD)] (Rinella et al., 2023). The term MASLD was introduced to reflect the close existence of steatosis and systemic metabolic dysfunction (Rinella et al., 2023). Although the definitions of NAFLD and MASLD differ, there is high concordance rates, with ∼95%–99% of people with NAFLD meeting the MASLD criteria (Hagstrom et al., 2024). For simplicity, throughout this review we will use the term MASLD.

Metabolic dysfunction‐associated steatotic liver disease encompasses a spectrum of liver pathologies beginning with steatosis, defined histologically as the presence of intracellular TG in >5% of hepatocytes, or >5.6% when assessed by proton MRI or spectroscopy (Fabbrini et al., 2009), through to metabolic‐associated steatohepatitis, advanced fibrosis and more severe liver disease (i.e., cirrhosis). The clinical burden of MASLD is not restricted to liver‐related complications and includes other extrahepatic manifestations of some cancers, chronic kidney disease and cardiovascular disease (Targher et al., 2024). Mendelian randomization studies have identified causally that steatotic liver disease can actively contribute to the development of cardiovascular disease (Ren et al., 2023), which is the leading cause of mortality for people with MASLD (Younossi et al., 2023, 2024).

Metabolic dysfunction‐associated steatotic liver disease is the most prevalent form of liver disease worldwide (Kalligeros et al., 2023), with a greater prevalence observed in those who are overweight and obese (Riazi et al., 2022) and in individuals with T2D (Younossi et al., 2024). Several studies have reported that MASLD and T2D frequently coexist and act synergistically to increase the risk of adverse hepatic and extrahepatic outcomes (Mantovani et al., 2020). The relationship between MASLD and T2D is complex and bidirectional, with the presence of one being a risk factor for the other. MASLD might precede or promote the development of T2D, and the risk of developing T2D parallels the severity of MASLD (Hazlehurst et al., 2016). Although there is no approved pharmacotherapy for the treatment of MASLD, several anti‐hyperglycaemic agents used to treat T2D (Glucagon‐like peptide‐1 (GLP‐1) receptor agonists, incretin receptor co‐agonists and sodium‐glucose cotransporter‐2 inhibitior (SGLT2i)) have been shown in phase 2 and 3 clinical trials to exert beneficial effects on MASLD and have cardioprotective effects, as reviewed in detail elsewhere (Mantovani et al., 2022; Targher et al., 2021).

## 3 INVESTIGATING INTRAHEPATIC TRIGLYCIERIDE METABOLISM in vivo IN HUMANS

Direct assessments of the human liver (by measurements of arteriovenous difference) are seldom performed owing to the inaccessibility of the portal vein; thus, the mechanisms that underpin disease aetiology and progression remain to be elucidated fully. Insight into molecular differences that might be involved in disease aetiology came from liver biopsies. Such methods are invasive and represent a single time point, which prevents insight into the aetiology and progression of MASLD.

**FIGURE 1:**
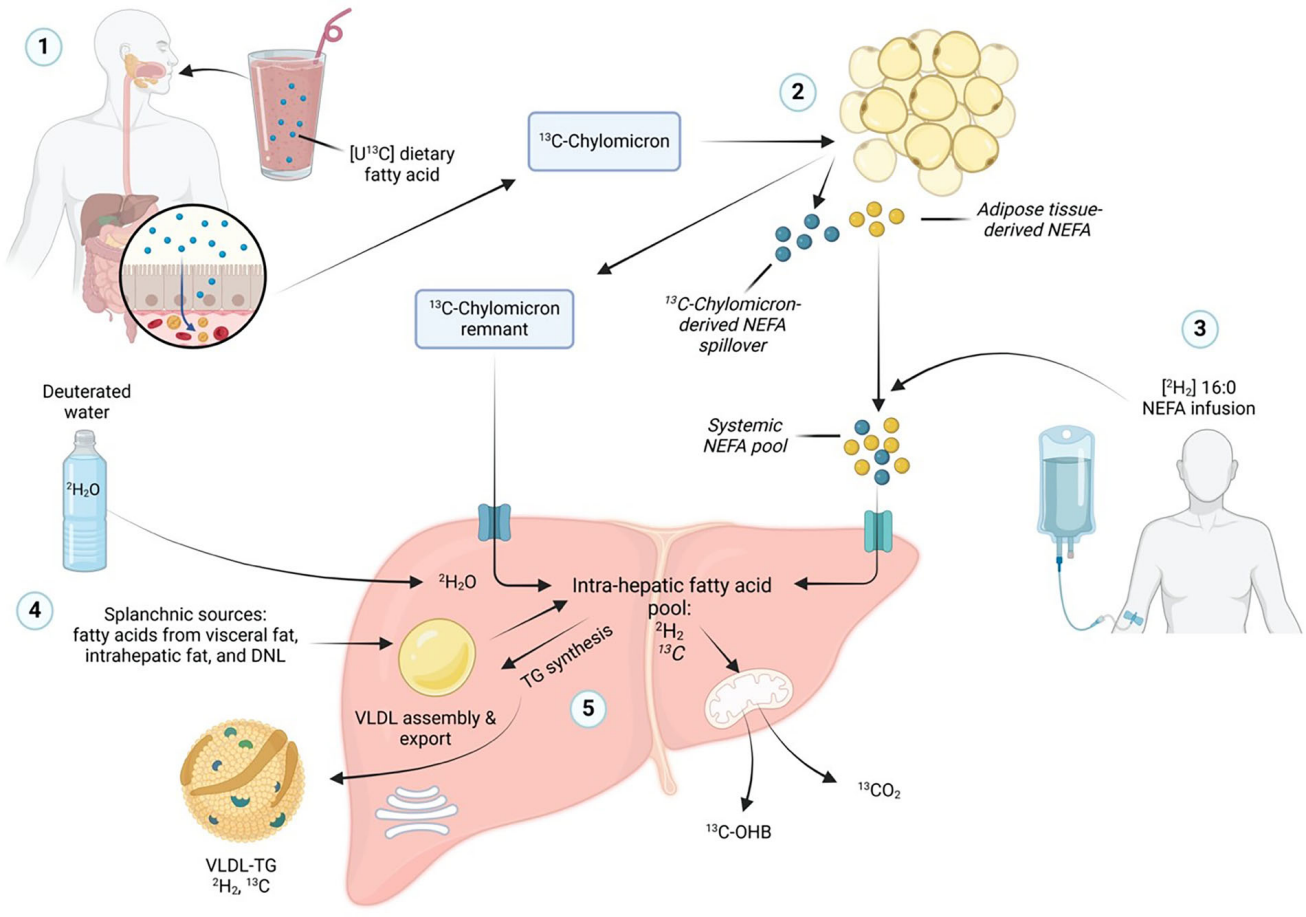
The ins and outs of hepatic FA metabolism using stable‐isotope tracer methodology. (1) The ingestion of a meal labelled with a [U‐^13^C]FA (e.g., [U‐^13^C]palmitic acid) allows for the tracing of exogenous fats. The [U‐^13^C]FA is incorporated into chylomicrons in the enterocyte and appears in systemic circulation as TG‐rich ^13^C‐chylomicrons, (2) where it is quickly hydrolysed by adipose tissue lipoprotein lipase, creating ^13^C‐chylomicron remnants that are taken up by the liver. Not all FAs from the hydrolysis of TG‐rich ^13^C‐chylomicrons are taken up across adipose tissue, causing a spillover of ^13^C‐NEFAs that appear in the systemic plasma NEFA pool. (3) Endogenous pathways are labelled using an intravenous infusion of [^2^H_2_]palmitate complexed with human albumin, which equilibrates with the systemic plasma NEFA pool to allow for the tracing of the whole‐body rate of appearance of NEFAs. Plasma NEFAs are taken up by the liver and introduced to the intrahepatic NEFA pool. (4) Given that water is used in the synthesis of FAs, the consumption of deuterated water (^2^H_2_O) is used to calculate the fractional synthesis of FA *de novo*. The ^2^H_2_O equilibrates with the total body pool, and ^2^H labels NADPH that is subsequently used for the formation of fatty acyl‐chains during the process of DNL. (5) Depending on nutritional state and metabolic health (i.e., insulin resistance, liver fat content), intrahepatic FAs are partitioned towards oxidation or are esterified to form TGs and are incorporated into VLDL and secreted into circulation, or stored within the hepatocyte. Abbreviations: CO_2_, carbon dioxide, DNL, *de novo* lipogenesis; FA, fatty acid; NEFA, non‐esterified fatty acid; TG, triglyceride; VLDL, very‐low density lipoprotein; OHB, 3‐hydroxybutyrate.

An alternative approach is to use surrogate markers of liver fat metabolism, including plasma concentrations of VLDL‐TG and 3‐hydroxybutyrate, which offer some insight into hepatic FA esterification and oxidation pathways, respectively (Donnelly et al., 2005; Havel et al., 1970; Kotronen et al., 2009). A more nuanced approach that provides the opportunity to trace the fate of specific FA sources through hepatic esterification and oxidation pathways involves the use of radioactive or stable‐isotope tracers (Figure 1) (Donnelly et al., 2005; Havel et al., 1970; Hodson et al., 2007; Luukkonen et al., 2018; Miles et al., 2004; Parry et al., 2020; Sunny et al., 2011), as reviewed by Umpleby (2015). Radio‐isotopes have been used for the *ex vivo* labelling of VLDL‐TG and allow the tracing of the fate of these particles in vivo (Gormsen et al., 2006). Radio‐tracers, in combination with positron emission tomography (PET), allow investigation of the FA metabolism within the splanchnic bed, while the simultaneous quantification of blood flow allows for calculation of flux rates (Iozzo et al., 2010; Risikesan et al., 2024). Given that the liver receives an influx of nutrients from multiple FA sources and that humans spend a large proportion of the day in the postprandial state (McQuaid et al., 2011), the use of stable‐isotope tracer methodology has provided the opportunity to trace the fate of these through different hepatic pathways (Figure 1).

## 4 FATTY ACID DELIVERY TO THE LIVER

### 4.1 Plasma NEFAs

There is a constant delivery of NEFAs to the liver, with the largest contribution deriving from the intracellular lipolysis of subcutaneous and visceral adipose tissue TG, while the FAs liberated by the hydrolysis of dietary TG carried in chylomicrons (chylomicron‐derived spillover NEFAs) represent a smaller contribution (Piche et al., 2018). Lipolysis of adipose tissue TG is regulated by insulin, which inhibits the activity of adipose TG lipase and hormone‐sensitive lipase. Therefore, plasma concentrations of NEFAs are greatest in the postabsorptive state when insulin levels are low and achieve their nadir after consumption of a mixed test meal (McQuaid et al., 2011). In contrast, intestinally derived chylomicrons carry dietary TG into systemic circulation and increase over the course of a postprandial period until they peak 2–4 h after ingestion of a mixed meal (Barrows et al., 2005; Hodson et al., 2007; McQuaid et al., 2011; Piche et al., 2018). The TG content of chylomicrons is quickly (∼5 min) hydrolysed by lipoprotein lipase (LPL) anchored on the endothelial surface of adipose tissue, creating remnant particles. Although the majority of liberated FAs are taken up in adipose tissue, some spill over into the systemic NEFA pool and can be taken up by the liver (Miles et al., 2004) (Figure 1). During the nadir in postprandial NEFA concentrations, we and others have demonstrated that chylomicron‐derived spillover NEFAs can constitute ∼50% of the total plasma NEFA pool (Barrows et al., 2005; McQuaid et al., 2011; Nelson et al., 2013; Piche et al., 2018).

Elevations in plasma NEFAs are observed in some, but not all, individuals with obesity and insulin resistance (Karpe et al., 2011) and are often suggested to be attributable to increased adipose tissue mass. However, when expressed per unit of fat mass, the rate of appearance of NEFAs across the fasting and postprandial states has been shown to be lower in obese compared with lean individuals (McQuaid et al., 2011), with release of NEFAs from subcutaneous adipose tissue (per 100 g of tissue) decreasing with increasing adiposity (Frayn & Humphreys, 2012; Karpe et al., 2011). Similar findings were observed when plotting the rate of appearance of NEFAs against fasting insulin concentrations (Karpe et al., 2011), suggesting that obese individuals might downregulate adipocyte lipolysis to aid the normalization of plasma NEFA concentrations (McQuaid et al., 2011).

Adipose‐derived NEFAs are the predominant source of FAs used for TG synthesis within the liver in both the fasting and postprandial states, despite there being a marked suppression in systemic NEFA concentrations in the postprandial state (Donnelly et al., 2005; Hodson et al., 2007, 2010; Jacome‐Sosa & Parks, 2014; Lambert et al., 2014; McQuaid et al., 2011; Parry et al., 2020; Pramfalk et al., 2016). The relative contribution of FAs from adipose tissue to VLDL‐TG differs between lean and obese individuals and between those with and without MASLD (Fabbrini et al., 2008; Hodson et al., 2007, 2010; Umpleby et al., 2017), which might be because these individuals have a greater contribution of FAs from splanchnic‐derived FA sources (i.e., IHTG, visceral adipose tissues and DNL) to VLDL‐TG (Hodson et al., 2007, 2010; Nelson, Basu, Johnson, et al., 2007). In the fasting state, visceral adipose tissue lipolysis is estimated to account for ∼10%–30% of hepatic FA delivery (Nelson, Basu, Johnson, et al., 2007; Nielsen & Jensen, 2024; Nielsen et al., 2004; Risikesan et al., 2024), the amount of which is proportional to total visceral fat mass (Nielsen et al., 2004). Obese individuals also demonstrate a greater splanchnic uptake of FAs (Nielsen & Jensen, 2024).

### 4.2 Dietary fat

Newly secreted chylomicrons carrying dietary TG are released into lymphatic channels and enter the systemic circulation via the thoracic duct. Evidence from a rat model has suggested that only half of the chylomicron TG content is hydrolysed by LPL and taken up by adipose tissue during the process of remnant formation (Hultin et al., 1996). This is appreciable, because a typical chylomicron particle contains ∼10^6^ molecules of TG (Frayn et al., 2006). Chylomicron remnants in the systemic circulation can be taken up by the liver via either the LDL receptor or LDLR‐related protein 1, and once in the liver, are hydrolysed by lysosomes to release FAs (Hodson & Gunn, 2019).

The overall contribution of chylomicron‐derived TG to IHTG synthesis remains unclear and will, to a degree, be dependent on the frequency and amount of dietary fat consumed and the phenotype of the individual. Studies tracing the fate of dietary fat over 12–24 h have found that the relative contribution of FAs from chylomicron remnants compared with FAs derived from chylomicron spillover was greater (∼15% compared with ∼10%, respectively) in VLDL‐TG (Barrows & Parks, 2006; Donnelly et al., 2005). Moreover, individuals with insulin resistance and/or obesity can have a greater flux of chylomicron‐derived FAs to the liver owing to a reduction in chylomicron TG uptake across adipose tissue (McQuaid et al., 2011). We have previously observed an inverse association between insulin resistance and chylomicron‐derived spillover NEFAs (Piche et al., 2018), which is consistent with the observation that the expression of adipose tissue LPL (and other FA trafficking genes) is downregulated in obese compared with lean individuals (McQuaid et al., 2011). The reduced extraction of dietary fat across adipose tissues can result in TG‐rich chylomicron remnants that can be taken up into the liver and provide a substrate for VLDL‐TG production or storage in cytosolic lipid droplets.

### 4.3 Hepatic uptake of FAs

Systemic FAs are taken up via transport proteins, including fatty acid transport proteins (FATPs), fatty acid binding protein, fatty acid translocase (CD36) and caveolins. In health, FATP2 and FATP5 are the predominant FATPs in the human liver, with CD36 being expressed in much lower levels (Miquilena‐Colina et al., 2011), whereas liver CD36 protein expression is upregulated in patients with MASLD compared with control subjects (Greco et al., 2008; Miquilena‐Colina et al., 2011). This is, in part, supported by studies in humans that have used PET coupled with computed tomography (PET/CT) in combination with labelled palmitate, demonstrating that hepatic FA uptake is greater in individuals with obesity (Immonen et al., 2018; Iozzo et al., 2010). Although FA transporters might exert a role in controlling hepatic FA flux, their role in the development of MASLD is unclear.

## 5 INTRAHEPATIC FATTY ACID PARTITIONING

### 5.1 Intrahepatic FA synthesis

*De novo* lipogenesis uses acetyl‐CoA as a carbon source for the synthesis of *de novo* FAs from non‐lipid precursors. Briefly, acetyl‐CoA is carboxylated by acetyl‐CoA carboxylase to form malonyl‐CoA, which undergoes elongation by fatty acid synthase to form the 16‐carbon FA, palmitate. Although palmitate (palmitoyl‐CoA) is often considered the primary product of DNL, it can be elongated to form stearate (18:0), and both palmitate and stearate can be desaturated further to form palmitoleate (16:1) and oleate (18:1), respectively (Hodson & Fielding, 2013). Once FAs are synthesized, elongated and desaturated, they can be esterified by the glycerophosphate backbone supplied by carbohydrate metabolism to form TG.

*De novo* lipogenesis is under tight transcriptional regulation, primarily by sterol regulatory element binding protein 1c (SREBP1c) and carbohydrate response element binding protein (ChREBP). Typically, the contribution of DNL‐derived FAs to VLDL‐TG is elevated in the postprandial state (Barrows & Parks, 2006; Timlin & Parks, 2005), owing to the upregulation of ChREBP and SREBP1c induced by increasing glucose and insulin concentrations (Hodson & Gunn, 2019). Insulin stimulates the upregulation of SREBP1c, which promotes the expression of lipogenic genes, including fatty acid synthase and acetyl‐CoA carboxylase (Hodson & Gunn, 2019). Likewise, in response to increased blood glucose concentrations, ChREBP stimulates the upregulation of lipogenic genes and drives an increase in genes involved in glycolytic flux and gluconeogenesis (Hodson & Gunn, 2019). Given that the transcriptional regulation of DNL is mediated, in part, by insulin, hyperinsulinaemia has been reported to be closely associated with increased DNL (Pramfalk et al., 2016), independent of body mass index (Schwarz et al., 2003).

In comparison to the systemic delivery of FAs to the liver, the quantitative contribution of DNL to whole‐body TG synthesis is low (Donnelly et al., 2005; Lambert et al., 2014). In healthy individuals, DNL is suggested to produce ∼1–2 g/day of newly synthesized lipids (Hellerstein, 1999), and even after carbohydrate overfeeding, which markedly upregulates DNL, the contribution of *de novo* FAs to VLDL‐TG is <5 g/day (Schwarz et al., 1995). Nonetheless, when DNL is upregulated, malonyl‐CoA inhibits carnitine palmitoyltransferase 1, leading to reduced transport of fatty acyl‐CoAs into the mitochondria. Thus, when DNL is upregulated constitutively, as seen in people with insulin resistance (Pramfalk et al., 2016) and MASLD (Lambert et al., 2014; Smith et al., 2020), this can lead to reduced FA oxidation (Pramfalk et al., 2016). Although the contribution of DNL‐derived FAs to VLDL‐TG is suggested to be between 15% and 38% in individuals with MASLD and between 1% and 10% in those without MASLD (Lambert et al., 2014), it remains unclear whether an upregulation in hepatic DNL is a cause or a consequence of IHTG accumulation. Indeed, DNL is elevated in individuals defined as insulin resistant (Pramfalk et al., 2016; Smith et al., 2020) and is purported to be a driver of IHTG accumulation (Lambert et al., 2014), although this has yet to be demonstrated conclusively in humans.

### 5.2 Oxidation and ketogenesis

Within the liver, FAs can be partitioned towards mitochondrial oxidation pathways, and circulating 3‐hydroxybutyrate concentrations are used as a surrogate marker for hepatic FA oxidation. In metabolically healthy individuals, the delivery of FAs from adipose tissue to the liver is a key regulator of β‐oxidation and ketogenesis, and the postprandial decrease in adipose tissue TG lipolysis markedly supresses ketogenesis. The transcriptional factor peroxisome proliferator‐activated receptor alpha (PPAR‐α) is the primary regulator of β‐oxidation, with FAs and glucagon upregulating PPAR‐α activity and β‐oxidation, whereas insulin has a suppressive effect.

The direct quantification of hepatic mitochondrial oxidation in vivo in humans is complex and challenging to do during dynamic conditions (i.e., after feeding). Studies providing orally labelled or infused ^13^C‐labelled tracers with the collection of expired breath samples for ^13^CO_2_ levels offer a marker of whole‐body FA oxidation (Figure 1). Measuring the incorporation of ^13^C in plasma 3‐hydroxybutyrate provides the opportunity to trace whether adipose‐derived FAs (intravenously administered ^13^C‐FAs) or exogenous FAs (orally administered ^13^C‐FAs) have gone through the ketogenic pathway. For instance, by incorporating uniformly labelled FAs (e.g., [U‐^13^C]palmitate or [U‐^13^C]linoleate) into a test meal, we previously showed that the whole‐body oxidation of dietary FAs is affected by both FA composition and participant sex (Parry et al., 2021). Others have taken an alternative approach by infusing multiple ^13^C‐labelled tracers and assessing the ^13^C isotopomer analysis of plasma by NMR to determine hepatic mitochondrial flux in overnight‐fasted individuals with and without liver fat (Sunny et al., 2011). The authors found that the mitochondrial fat metabolism pathways (i.e., the tricarboxylic acid cycle, anaplerosis) were elevated in those defined as having high IHTG in comparison to those with low IHTG, with no difference between groups in 3‐hydroxybutyrate turnover (Sunny et al., 2011). Using isotopomer analysis, Fu et al. (2023) demonstrated that fasting DNL in MASLD subjects was associated with lower hepatic ketogenesis and β‐oxidation, but an increased tricarboxylic acid cycle flux. We have previously found a strong association between hepatic DNL and ketogenesis in individuals defined as normoinsulinaemic, with no association in those defined as hyperinsulinaemic (Pramfalk et al., 2016). The lack of association we observed might be explained, in part, by the duration an individual had been hyperinsulinaemic, because observations in a diet‐induced mouse model of obesity and insulin resistance noted a progressive adaptation of hepatic ketogenesis during high‐fat feeding, resulting in increased hepatic fat oxidation, suggesting that mitochondrial fat oxidation is stimulated rather than impaired during the initiation of hepatic insulin resistance (Sunny et al., 2010). More recently, state‐of‐the‐art positional isotopomer NMR tracer analysis (PINTA) has been used to quantify hepatic mitochondrial oxidation (Luukkonen et al., 2023; Petersen et al., 2024). The use of PINTA has enabled the non‐invasive insight into hepatic mitochondrial function in people with and without disease (Luukkonen et al., 2023; Petersen et al., 2024).

Early work by Havel et al. (1970) demonstrated that, despite a similar FA delivery rate to the liver, there were differences in the partitioning of FAs into the respective pathways between obese, hyperlipidaemic and normolipidaemic individuals. By infusing 1‐^14^C‐palmitate in the postabsorptive state, the authors revealed that in normolipidaemic individuals, around two‐thirds of FAs entering the liver were partitioned towards oxidation, rather than esterification pathways, whereas the proportion of FAs entering oxidation and esterification pathways was similar in hyperlipidaemic individuals.

More recent work has reported that increasing MASLD severity is associated with a reduction in hepatic FA oxidation (Moore et al., 2022), suggesting that impairments in FA oxidation might contribute to the pathogenesis of metabolic dysfunction. Petersen et al. (2024) demonstrated that hepatic mitochondrial oxidation, assessed by PINTA, was similar between overweight individuals without hepatic steatosis and those with MASLD or metabolic‐associated steatohepatitis, and subjects in all groups were similarly responsive to glucagon. The discrepancy in findings might be attributable to the methods used or differences in participant genotype (Luukkonen et al., 2023).

### 5.3 Intrahepatic lipid hydrolysis

Within the liver, two central processes, lipolysis and autophagy, have been proposed to mediate the breakdown of TG stored within lipid droplets. Lipolysis is mediated by hepatic adipose TG lipase, and the expression of adipose TG lipase, in addition to its cofactor CG1‐58, has been reported to be downregulated in liver biopsies of MASLD patients compared with control subjects (Kato et al., 2008). A highly selective form of lipid droplet‐specific autophagy (known as lipophagy) occurs when lipid droplets are hydrolysed in autolysosomes to release FAs into the cytosol; lipophagy has been suggested to impact IHTG dynamics (Singh et al., 2009). Although the mechanisms regulating lipophagy are not well understood, a reduction in protein markers of autophagy has been reported in liver biopsies from MASLD patients compared with healthy control subjects (Rada et al., 2020), and these findings are supported by preclinical rodent MASLD models that demonstrate impairments in autophagic flux (Rada et al., 2020). The relative contribution of these pathways, and whether lipolysis and lipophagy occur simultaneously or independently, remain unclear. However, findings from murine models have shown that a decrease in either pathway drives IHTG accumulation (Singh et al., 2009), suggesting a mechanistic link between dysfunction of either pathway and IHTG accumulation.

### 5.4 Fatty acid esterification

Fatty acids are delivered to the liver via the systemic circulation and via hepatic portal delivery, with the latter largely reflecting mobilization of FAs from the visceral compartment (Figure 1). Within the hepatocyte, newly delivered FAs are activated by acyl‐CoA synthetases to form fatty acyl‐CoAs. Alternatively, the fatty acyl‐CoA pool can originate from the uptake of lipoprotein remnants and their catabolism within lysosomes, or those synthesized from DNL. Depending on the nutritional and hormonal state, fatty acyl‐CoA will be directed towards mitochondrial oxidation or esterification for glycerolipid synthesis, with the final step of TG synthesis being catalysed by diacylglycerol acyltransferase (DGAT) enzymes (Hodson & Gunn, 2019). In hepatocytes, there are two forms of DGAT: DGAT1 esterifies exogenous FAs, whereas DGAT2 uses DNL‐derived diacylglycerols. It has been suggested that TG from DGAT2 is preferentially partitioned to endoplasmic reticulum pools for immediate secretion in VLDL particles, whereas TG from DGAT1 might be more directed towards storage in lipid droplets (McFie et al., 2021). TG synthesis can also occur via the hydrolysis and re‐esterification of TG from the intrahepatic pool, which involves the addition of a fatty acyl‐CoA to a preformed monoacylglycerol (Hodson & Gunn, 2019).

## 6 SECRETION OF HEPATIC FATTY ACIDS

Fatty acids and cholesterol esters that are esterified to TG can be secreted from the liver in VLDL (Boren et al., 2022). The formation of VLDL begins in the endoplasmic reticulum, where TGs are transferred to a nascent apolipoprotein B‐100 particle by microsomal TG transfer protein, forming a primordial VLDL_2_ particle with a small TG core, with a second lipidation step being required for formation of a mature VLDL_1_ particle (Boren et al., 2022). Although the exact steps underlying and regulating this process remain unclear, it is likely to involves an intrahepatic lipolysis‐re‐esterification cycle that uses luminal lipid droplets and fusion of VLDL_2_ as a substrate pool. In humans, carboxylesterase enzymes 1 and 2 are the most well‐defined lipases associated with VLDL assembly and are hypothesized to hydrolyse luminal lipid droplets for the second step in VLDL_1_ lipidation (Ruby et al., 2017). Mature VLDL_1_ particles undergo vesicle‐mediated transfer to the Golgi apparatus before being released into the circulation via specialized transport vesicles (Boren et al., 2022).

Although both substrate availability and insulin appear to have a major influence on VLDL‐TG production, particularly VLDL_1_ particles, the formation and secretion of VLDL_2_ does not appear to be dependent on FA availability (Karpe & Hamsten, 1995) or on insulin (Malmstrom et al., 1998). Elevation in insulin, as seen postprandially, suppresses the release of VLDL_1_ by both direct and indirect mechanisms (i.e., anti‐lipolytic and reduced NEFA flux to the liver) (Malmstrom et al., 1998). For a given insulin concentration, females secrete fewer moles of VLDL‐TG than males (Mittendorfer et al., 2016). The observed differences in VLDL‐TG secretion rate between men and women are proposed to be attributable to differences in the incorporation of systemic FAs into VLDL‐TG (Nielsen et al., 2003), rather than potential differences in DNL (Pramfalk et al., 2015). It is proposed that the direct suppression of VLDL_1_ production by insulin in the postprandial state might reflect a compensatory mechanism in response to the increased presence of chylomicrons in the circulation, which compete with VLDL‐TG for LPL activity (Boren et al., 2022). We have previously demonstrated in healthy adults that the fractional extraction of TG from VLDL across subcutaneous abdominal adipose tissue is around four to five times less in comparison to chylomicron TG extraction, suggesting that chylomicron TGs are the preferred substrate for lipoprotein lipase (Bickerton et al., 2007).

Excess TG in the endoplasmic reticulum increases the number of nascent VLDL particles that can enter the assembly and secretion pathway, and favours VLDL_1_ formation (Boren et al., 1990). Individuals with elevated IHTG accumulation exhibit an overproduction of VLDL, particularly VLDL_1_ (Adiels et al., 2006), which might occur to protect the liver from further TG overload. By studying body mass index‐ and body fat‐matched individuals, (Fabbrini et al., 2008) observed that the postabsorptive secretion rate of TG‐rich VLDL_1_ particles was twofold greater in individuals with an elevated (∼22.7%) IHTG content, in comparison to those with normal liver fat levels (∼3.4% IHTG). Likewise, Umpleby et al. (2017) reported that people with MASLD secrete larger and more TG‐rich VLDL particles, in comparison to the VLDL particles secreted from individuals without MASLD. There appears to be a limit in VLDL secretion, with a plateau in VLDL_1_ secretion rates being observed at IHTG levels of >10% (Fabbrini et al., 2008). Although it is unclear what factors limit the secretion of VLDL‐TG in humans, evidence from transgenic mice that overexpress SREBP‐1a and develop steatosis demonstrates that very large VLDL particles can exceed the diameter of the sinusoidal endothelial pores, resulting in their accumulation as IHTG (Horton et al., 1999). Moreover, when steatosis accounts for >30% of liver volume, the genes encoding microsomal TG transfer protein and apolipoprotein B are downregulated (Higuchi et al., 2011), thereby potentially impeding the removal of synthesized lipids from the liver. This is also observed in carriers of a mutation in the Transmembrane 6 superfamily 2 gene, where there is a reduction in VLDL secretion (Sliz et al., 2018), causing lipids to be partitioned towards their accumulation in intracellular lipid droplets, rather than their export.

In humans, kinetic studies have demonstrated that the suppressive effect of insulin on VLDL_1_ secretion is diminished in individuals with elevated IHTG content (Adiels et al., 2007). The mechanisms for the direct action of insulin on VLDL_1_ remain to be elucidated but might be related to hepatic insulin resistance and the accumulation lipid species in cytosolic lipid droplets that impede the final lipidation step (Boren et al., 2022). There are also clear sex differences in VLDL‐TG metabolism, such that for any given degree of adiposity, adult males have higher plasma VLDL‐TG concentrations, compared with females, which is largely attributable to an increase in VLDL‐TG secretion (Mittendorfer et al., 2016). Moreover, elevations in plasma VLDL‐TG concentrations in obese males compared with their lean counterparts result from the over‐secretion of VLDL_1_ particles, whereas the reduced clearance of VLDL_1_ particles is the primary determinant of elevated systemic VLDL‐TG concentrations in obese females (Mittendorfer et al., 2016). The overproduction and impaired clearance of VLDL_1_ are considered to be the underlying factors in the development of hypertriglyceridaemia and atherogenic dyslipidaemia in people with increased IHTG content (Taskinen et al., 2011).

## 7 EFFECT OF DIET ON INTRAHEPATIC FATTY ACID ACCUMULATION AND PARTITIONING

Several studies have examined the effects of dietary macronutrient manipulation on IHTG responses, as reviewed by Yki‐Jarvinen et al. (2021) with only a limited number of studies investigating the mechanisms by which diet or macronutrient composition can affect the processes underlying IHTG accumulation. For example, Luukkonen et al. (2018) demonstrated, in a 3‐week overfeeding study, that the ingestion of a hypercaloric (+1000 kcal/day) diet rich in saturated fat induced greater accumulation of IHTG compared with a hypercaloric diet enriched in free sugars or unsaturated fats (+1000 kcal/day). The authors reported that saturated fat impaired the ability of insulin to suppress adipocyte lipolysis during an euglycaemic–hyperinsulinaemic clamp with the co‐infusion of [^2^H_5_]glycerol, in comparison to baseline responses, whereas consumption of a diet enriched in free sugars or unsaturated fat had minimal effects on lipolysis. DNL was markedly increased after the high‐sugar diet, but not after the saturated or unsaturated fat diets (Luukkonen et al., 2018). Rosqvist et al. (2014, 2019) found that a hypercaloric diet enriched in saturated fat induces IHTG accumulation to a greater extent than a hypercaloric diet enriched in polyunsaturated fat in both lean and overweight individuals. The authors demonstrated that the rate of hepatic FA uptake, measured by PET, was not affected by dietary fat composition (Rosqvist et al., 2019), implying that additional factors beyond hepatic FA uptake might play a more pathophysiological role in the regulation of IHTG accumulation. Limited evidence in humans suggests that polyunsaturated FAs are partitioned preferentially to enter oxidation pathways compared with saturated FAs (Parry et al., 2021; Sahini & Borlak, 2014; Schmidt et al., 1999; Srnic et al., 2024). We have recently demonstrated that even when hepatocellular metabolism is shifted towards esterification by an upregulation of DNL, polyunsaturated FAs still preferentially enter oxidation pathways compared with saturated FAs (Srnic et al., 2024).

Our previous work has reported that the adverse effects of a diet high in saturated fat on IHTG accumulation are also evident in the absence of meaningful body weight gain in adults free from metabolic disease (Parry et al., 2020). We found that the ingestion of a eucaloric saturated fat‐rich diet for 4 weeks increased IHTG content to a greater extent than consumption of a sugar‐rich diet, with no observed differences in whole‐body FA oxidation, the rate of appearance of NEFA, DNL or the contribution of FA sources in VLDL‐TG between diets (Parry et al., 2020). The reason why a diet rich in saturated fat had such a profound effect on IHTG remains unclear, although the overfeeding of dietary saturated fats consistently augments an inflammatory response and stimulates the synthesis of ceramides (Luukkonen et al., 2018; Rosqvist et al., 2019), processes that might favour IHTG accumulation. For example, Rosqvist et al. (2019) demonstrated that the differential effects of a diet enriched in either saturated fat or polyunsaturated fat on IHTG content are abolished after adjustment for changes in C16 serum ceramides (proposed to reflect ceramides of hepatic origin), supporting the role of ceramide synthesis in dietary saturated fat‐mediated IHTG accumulation. Given that the latter study was performed in a hypercaloric manner [weight gain of ∼2.5 kg (3%)], whether such effects are observed during eucaloric feeding remains unclear and warrants further investigation.

### 7.1 Effect of nutrient composition on VLDL‐TG kinetics

There is a strong association between VLDL‐TG concentrations and IHTG content (Adiels et al., 2006; Donnelly et al., 2005), and the VLDL‐TG FA composition is purported to reflect the composition of IHTG (Donnelly et al., 2005). VLDL‐TG concentrations reflect a composite of secretion and clearance, and perturbations in VLDL secretion or clearance, or both, can have marked effects on systemic VLDL‐TG concentrations (Mittendorfer et al., 2016). Despite this, the inter‐relationship between diet and VLDL kinetics has seldom been studied. In a 12‐week randomized crossover study involving men with (*n* = 11) and without (*n* = 14) MASLD, Umpleby et al. (2017) examined the effects of a hypercaloric diet of low (6% total energy) or high (26% total energy) sugar content on postabsorptive lipoprotein kinetics, as measured by stable‐isotope tracing. The authors found that the ingestion of a diet enriched in free sugars increased production of TG‐rich VLDL_1_ compared with the low‐sugar diet in the healthy participants, which was accompanied by an increase the contribution of DNL‐derived FAs to VLDL_1_‐TG. In participants with MASLD, the high‐sugar diet did not affect DNL or the secretion or clearance rate of VLDL_1_, suggesting that they were already working at maximal capacity. Despite comparable increases in body weight after each diet (∼2 kg), IHTG content increased approximately twofold after the high‐sugar diet in both MASLD and control groups (Umpleby et al., 2017). In contrast, when adults with hypercholesterolaemia were fed a standardized eucaloric diet for 6 weeks (52%/32%/16% total energy from carbohydrates, fats and protein, respectively) that differed in FA composition of saturated fat/monounsaturated fat (14%/7% and 7%/14% of energy intake, respectively), the production and clearance rate of VLDL_1_ or VLDL_2_ particles was not affected (Gill et al., 2003). Whether similar findings would be observed in diets that manipulate both the amount and the type of a macronutrient consumed (e.g., a high‐fat diet composed of saturated fats) is unclear.

### 7.2 Effect of meal frequency on IHTG accumulation

An aspect of human dietary patterns that is often overlooked in physiological feeding studies is the frequency with which nutrients are consumed. In a small, comprehensive study, Koopman et al. (2014) demonstrated that both meal frequency and macronutrient composition can have profound effects on IHTG accumulation, independent of overall energy intake and change in body weight. The authors showed that consuming 40% excess energy from sugar‐sweetened beverages 2–3 h after each main meal resulted in a significant relative increase (+110%) in IHTG content over the 6‐week intervention period in lean males, whereas when the sugar‐sweetened beverages were consumed with the main meal they failed to affect IHTG. Furthermore, although snacking with a fat‐rich beverage also increased IHTG content (relative increase +40%) of participants, this effect was markedly smaller compared with what was observed after snacking with sugar‐sweetened beverages (Koopman et al., 2014). Although the mechanisms explaining these findings remain to be elucidated, it is possible that the repeated hepatic uptake of dietary sugars would result in the sustained upregulation in DNL and concomitant downregulation in hepatic FA oxidation, thereby maintaining hepatocellular metabolism towards FA esterification. This concept, in part, has been reported in bovine hepatocytes, with the overexpression of SREBP1c causing a reduced expression of apolipoprotein B‐100 and impaired microsomal TG transfer protein activity, leading to reduced VLDL synthesis and VLDL‐TG export and the subsequent deposition of IHTG (Li et al., 2014). Further studies designed to delineate how the frequency of nutrient intake affects the mechanisms underpinning IHTG accumulation are warranted.

## 8 CONCLUSION

**FIGURE 2:**
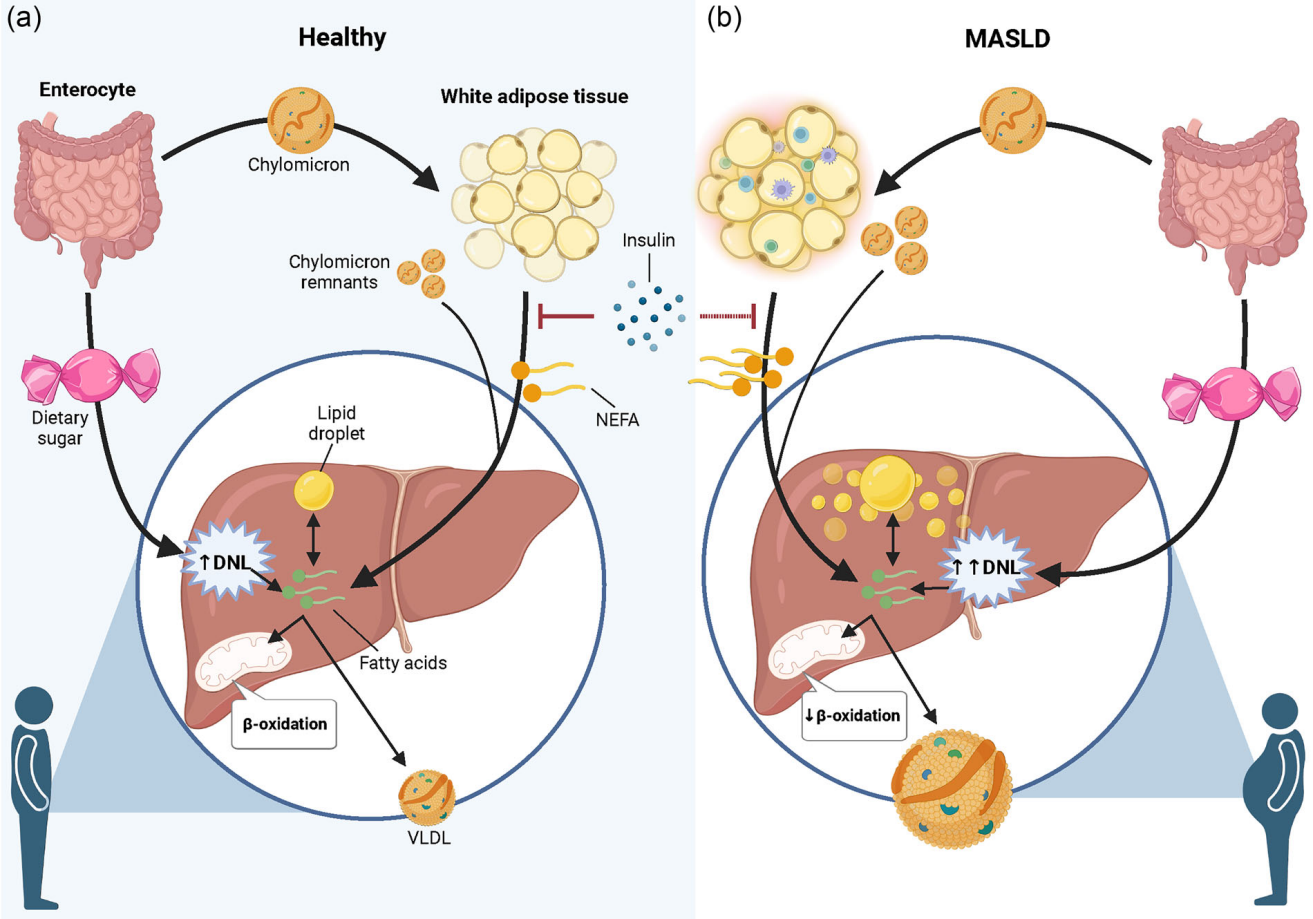
Hepatic FA partitioning in healthy individuals (a) and in those with MASLD (b). (a) When insulin concentrations are low, as seen in the fasting state, the hydrolysis of adipose tissue TG releases NEFAs that are taken up by the liver and mix with FAs from the cytosolic TG pool. Within the liver, FAs can be used for energy production via β‐oxidation or for TG synthesis. FAs esterified to TGs are either incorporated into VLDL and exported into the systemic circulation or partitioned to storage within cytosolic lipid droplets. Dietary fat is packaged into chylomicrons as TG in enterocytes and enters the circulation. Chylomicrons quickly undergo hydrolysis by adipose tissue lipolysis, liberating FAs to be taken up in adipose tissue, thereby creating chylomicron remnants, which are taken up by the liver. Some FAs will escape uptake across adipose tissue and appear in the systemic NEFA pool. Dietary sugars and other non‐lipid precursors can be used to form FAs in the liver via DNL. The secretion of insulin suppresses adipose tissue lipolysis and stimulates FA uptake across adipose tissue and increases DNL, thereby shifting the cellular metabolism of FA from oxidative to esterification pathways. (b) The production and secretion of large, TG‐rich VLDL particles is upregulated in the presence of elevated intrahepatic TG content owing to the increased delivery and uptake of NEFAs and chylomicron remnants, increased DNL and excessive TG storage in cytosolic lipid droplets. Abbreviations: DNL, *de novo* lipogenesis; FA, fatty acid; MASLD, metabolic dysfunction‐associated steatotic liver disease; NEFA, non‐esterified fatty acid; TG, triglyceride; VLDL, very‐low density lipoprotein.

Many factors that are involved in the regulation of intrahepatic delivery, synthesis, partitioning and export of FAs can individually or synergistically influence IHTG accumulation (Figure 2). Perturbations in hepatic FA metabolism have far‐reaching consequences and are closely associated with an increased risk of cardiometabolic disease, including MASLD and T2D.

It is well described that both dietary composition and the phenotype (e.g., adiposity and sex) of an individual have profound effects on IHTG accumulation; however, their interaction on liver metabolism in vivo remains poorly understood. Studies using stable‐isotope tracers have delineated some of the complexity of how the phenotype of an individual along with how dietary composition alter the regulation of intrahepatic FA metabolism. Evidence to date suggests that, independent of overall body weight gain, diets enriched in saturated fats or those defined by frequent free sugar intake drive IHTG accumulation, probably through several diverse but poorly elucidated mechanisms. Although it is well described that the intrahepatic metabolism of FA differs by saturation, there are only a handful of investigations exploring the mechanisms driving the effect of divergences in dietary fat composition on IHTG content in humans. Advancements in imaging techniques, complemented by in vitro and preclinical models designed to reflect aspects of metabolic disease (Deja et al., 2024), have the potential to elucidate the further underlying mechanisms responsible for the complex cascade of events that can lead to IHTG accumulation.

## AUTHOR CONTRIBUTIONS

Kieran Smith, Kaitlyn M. J. H. Dennis and Leanne Hodson all contributed equally to the conceptualization of this work. Kieran Smith wrote the original draft with contributions from Kaitlyn M. J. H. Dennis, and all authors critically reviewed and edited the manuscript. All authors provided approval of the final manuscript and agree to be accountable for all aspects of the work in ensuring that questions related to the accuracy or integrity of any part of the work are appropriately investigated and resolved. All persons designated as authors qualify for authorship, and all those who qualify for authorship are listed.

## CONFLICT OF INTEREST

None declared.
